# Physical Activity during Pregnancy: Impact of Applying Different Physical Activity Guidelines

**DOI:** 10.1155/2013/165617

**Published:** 2013-02-05

**Authors:** Katie M. Smith, Christina G. Campbell

**Affiliations:** Interdepartmental Graduate Program in Nutritional Sciences, Department of Food Science and Human Nutrition, Iowa State University, 220 MacKay Hall, Ames, IA 50011, USA

## Abstract

Multiple guidelines and definitions of physical activity (PA) have been used to study the benefits of activity during pregnancy. The different guidelines lead to a wide range of prevalence estimates and this has led to conflicting reports about activity patterns during pregnancy. A longitudinal study was conducted to assess PA using a pattern-recognition monitor for a 7-day period at week 18 (*n* = 55) and week 35 (*n* = 66) of pregnancy. The amount of activity performed and the number of women meeting six different PA guidelines were evaluated. Adherence to PA guidelines ranged from 5 to 100% and 9 to 100% at weeks 18 and 35, respectively. All women achieved the 500 MET-minute guideline and nearly all women accumulated ≥150 minutes of weekly moderate-vigorous physical activity (MVPA) at both time points. Only 22% and 26% participated in ≥3 sessions of MVPA lasting ≥30 minutes at both time points and this further declined to 5% and 9% when the guideline was increased to ≥5 sessions of 30 minutes. The amount of PA during pregnancy varied drastically depending on which guideline was used. Further research is warranted to clearly identify the patterns of activity that are associated with healthy pregnancy outcomes.

## 1. Introduction

Views on physical activity and exercise during pregnancy have taken on new meanings and implications throughout history. The importance of maternal physical activity dates as far back as the third century BC when Aristotle eluded to the difficulty endured during childbirth as a result of a sedentary maternal lifestyle [1]. However, society and expert opinions have not always supported the prenatal exercise since that time. For many years maternal, exercise was thought to harm the fetus or promote adverse pregnancy outcomes such as preterm delivery and fetal growth restriction or small for gestational age infants [2, 3]. In 1985, the American College of Obstetricians and Gynecologists (ACOG) published the first exercise guidelines for pregnant women. These included limitations on heart rate and duration, restricting heart rate to 140 beats per minute, and exercise to no more than 15 minutes at a time [4]. Furthermore, women that were inactive prior to pregnancy were not advised to begin an exercise program while pregnant. Considerable evidence was published regarding the safety of maternal exercise between the 1980s and early 1990s supporting the need for updated and revised exercise guidelines [3, 5, 6]. Consequently, ACOG responded in 1994 by eliminating the constraints on heart rate and exercise duration, stating that exercise can be done in moderation but not to exhaustion [7]. Finally, in 2002, ACOG issued a statement promoting the health benefits and safety of exercise in pregnancy for both previously active and inactive women (assuming medical clearance and no contraindications are present) [2]. These recommendations of 30 minutes or more of moderate exercise on most, if not all, days of the week were reaffirmed by ACOG in 2009 [2].

Most recently, physical activity recommendations for pregnant women were also included in the first ever Physical Activity Guidelines for Americans published in 2008 by the United States Department of Health and Human Services (DHHS) [8]. In this document, pregnant women (previously active and inactive) are encouraged to engage in at least 150 minutes of moderate-intensity aerobic activity each week. Women already doing regular activity of vigorous intensity may continue provided that they remain healthy and discuss their activity with their healthcare provider over time [8]. Recommendations for the nonpregnant population are very similar; however, they specifically state that the activity can be accumulated in minimum bouts of 10 minutes. The recommendation for shorter sustained bouts of activity transpired from a summary of experimental findings in nonpregnant adults suggesting that activity performed at a level of at least moderate intensity and sustained for at least 10 minutes at a time was as effective as single, longer bouts of activity in lowering chronic disease risk [9]. The Physical Activity Guidelines for all American adults, including pregnant women, encourage the activity to be spread throughout the week. However, specific minimum bouts of activity clarifying “what counts” towards meeting activity guidelines during pregnancy, such as the 10-minute bouts for nonpregnant adults, are not explicitly stated in the pregnancy guidelines for Americans [8]. Conversely, the 2011 Canadian Physical Activity Guidelines suggest that accumulating 150-minutes of weekly moderate- to vigorous-intensity aerobic physical activity in bouts of 10-minutes or more may be appropriate for pregnant women [10].

Previous studies have evaluated the prevalence of activity during pregnancy using multiple interpretations of the ACOG guidelines. Some studies have focused on accumulating at least 30 minutes of moderate-vigorous physical activity (MVPA) throughout the day [11, 12]. For example, McParlin et al. assessed the percentage of overweight and obese pregnant women accumulating at least 30 minutes of MVPA per day [11]. Their results revealed 63%, 62%, and 71% of women accumulating the recommended amount of activity in the 1st, 2nd, and 3rd trimester, respectively. Conversely, Chandonnet et al. compared the amount of accumulated MVPA performed by obese pregnant women to MVPA performed in at least 10-minute bouts [12]. Average total daily activity was drastically reduced by 66 minutes per day when only the activity that lasted for at least a 10-minute bout was counted. These two studies demonstrate that how the guideline is interpreted influences the number of women meeting the ACOG recommendations. As described, multiple guidelines have been used to assess levels of physical activity and exercise throughout pregnancy. The differences in guidelines have led to widely disparate estimates of the prevalence of pregnant women achieving physical activity guidelines during pregnancy—ranging from 3 to 78.4% [11, 13–20]. Furthermore, the use of multiple guidelines has contributed to conflicting evidence regarding the role of physical activity to improve certain pregnancy outcomes, such as healthy gestational weight gain, insulin sensitivity, and preeclampsia. To date, no study has specifically evaluated the impact of different physical activity guidelines on the reported patterns of physical activity using an objective assessment tool evaluated for use in pregnancy. Understanding the implications of the subtle, but distinct, differences between guidelines is an important consideration to explain the inconsistency of previous studies and to improve the reporting of physical activity during pregnancy. The purpose of this study was twofold: (1) to evaluate the difference in the minutes of physical activity that women participate in during pregnancy depending upon what guideline is used and (2) to compare the percentage of women that meet physical activity guidelines during pregnancy depending on what guideline is used. Data were evaluated for the second and third trimesters to demonstrate the impact of these guidelines across pregnancy.

## 2. Materials and Methods

### 2.1. Participants

Eighty-nine healthy pregnant women prior to 18-week gestation were enrolled for a larger longitudinal study analyzing the relationship between maternal exercise and fetal docosahexaenoic acid status. Participants were recruited via local obstetric clinics, fliers placed in town, and online and campus-wide emails. All women were screened to ensure they met the study's inclusion criteria (singleton pregnancy and maternal age of 18–45 years of age) and exclusion criteria (smoker or history of chronic disease) which was verified by each participant's primary obstetric medical provider. Due to the observational design of the study, no additional medical prescreens for exercise were needed. Nineteen women did not complete the study due to pregnancy complications or personal time constraints. Additionally, 13 other women at week 18 and 1 woman at week 35 had incomplete datasets, while 2 other women at week 18 and 3 women at week 35 had substantial off-body time (further described in “*Data processing*”); thus data were analyzed for 55 women at week 18 and 66 women at week 35. The protocol was approved by the campus' Institutional Review Board.

### 2.2. Study Design

Participants visited the research facility for 2 data collection periods lasting 7 days each at 18 and 35 weeks (±1 week) of pregnancy. During the week 18 appointment, participants provided written informed consent and completed a medical history questionnaire indicating their age, ethnicity, education, parity, height, pre-pregnancy weight, and due date. At the beginning of both data collection periods, participants were weighed without shoes using a Sunbeam analog scale (2008 Sunbeam Products, Inc., Boca Raton, Florida). A SenseWear Mini Armband monitor (Model Name: MF) (SWA) (BodyMedia, Inc., Pittsburgh, Pennsylvania) was then configured for each woman to quantify physical activity. The monitor was initialized according to her height, weight, age, and handedness and placed on her upper left arm per manufacturer's instructions. Participants were instructed to wear the monitor for the subsequent 7-day period, 24 hours a day except during any water submersion activities such as showering and swimming. Additionally, all the daily activity was to be documented in a provided physical activity record to confirm activity while the monitor was not worn (e.g., swimming and bathing). Staff members instructed each woman to participate in her normal daily activity and return the SWA and physical activity record to the research facility at the end of the 7-day data collection period. The amount of moderate and vigorous activity was assessed by the SWA except for activity endured during water submersion (e.g., swimming or water aerobics). Water activity was confirmed by the physical activity record. 

### 2.3. Physical Activity Armband

The validity of the SWA to predict energy expenditure in pregnant women has been previously assessed and correlated well with indirect calorimetry, *R* ^2^ = 0.86 [21]. The monitor is a pattern-recognition monitor with a triaxial accelerometer, heat-flux thermometers, a galvanic skin response sensor, and a skin temperature sensor. The SWA uses these sensors via the use of proprietary algorithms to predict the energy expenditure. The monitor records data in 1-minute epochs and provides a metabolic equivalent (MET) value for each minute of activity using the equation METS = kcal *·* hour^−1^ *·* kg^−1^. The raw SWA files were sent to the manufacturer (BodyMedia, Inc.) and processed with algorithm 5.2. 

### 2.4. Data Processing

SWA data files returned from the manufacturer were exported into Microsoft Office Excel 2007 (Microsoft, Redmond, WA,USA). Excel code was written to identify and categorize total minutes spent in moderate or vigorous activity. Previous research with the SWA in this population demonstrated an overestimation of energy expenditure by approximately 20% (*R* ^2^ = 0.86) [21]. To adjust for this overestimation, 20% was added to the standard MET thresholds for moderate (3–5.9 METs) and vigorous activity (≥6 METs) such that the SWA MET thresholds used in the current study were 3.6–7.1 METs (moderate) and ≥7.2 METs (vigorous). Similarly, total accumulated MET minutes were reduced by 20% (independently of categorizing METs into increased intensity thresholds). SWA data files were thoroughly reviewed to identify periods of nonwear time to ensure files were as close to 24-hours of wear time as possible. Two women did not wear the monitor at night. This particular nonwear time activity was confirmed to be spent sleeping by checking the physical activity record and subsequently this time was filled as sedentary time, equivalent to 0.95 METs [22]. Twenty-six women participated in water activities such as swimming or aqua aerobics. Nonwear time spent doing water activities was accounted for using corresponding MET values from the 2011 Compendium of Physical Activities [22]. To evaluate the remaining nonwear time, it was assumed that nearly 1 hour of self-care per day would result in approximately 500 minutes of nonwear time per week; thus files with more than 500 minutes per week of off-body time were deemed as noncompliant and excluded from the analysis for that data collection period. This excluded 2 files at week 18 and 3 files at week 35 as previously mentioned in paragraph 2.1. 

### 2.5. Physical Activity Guideline

The armband data files were processed and evaluated using six different physical activity guidelines. These guidelines were either previously used to define women that were exercising regularly throughout pregnancy [16, 23] or to assess prevalence of women meeting weekly physical activity recommendations during pregnancy [2, 7, 8, 11–15, 17–20, 24]. The guidelines included (1) 150-minutes of accumulated moderate-vigorous physical activity (MVPA) [2, 11, 17, 19], (2) 150 minutes of MVPA performed in periods of at least 10 minutes [12], (3) 150 minutes of MVPA performed for at least 10 minutes with 1 minute of vigorous activity equivalent to 2 minutes of moderate activity (M2VPA) [8], (4) at least 3 sessions of MVPA [7, 24] sustained for at least 30 minutes at a time [14, 16, 23], (5) at least 5 sessions of MVPA sustained for at least 30-minutes at a time [13, 15, 20], and (6) at least 500 MET minutes accumulated throughout the week [18]. Four of these guidelines included the use of a minimum bout of activity, 10 (guideline 2 and 3) or 30 minutes (guideline 4 and 5). When analyzing data for these 4 guidelines, interruptions of 1 or 2 minutes below the moderate intensity threshold within a 10-minute period were allowed, as has been previously reported with analysis of physical activity data in the nonpregnant population [25, 26]. 

The percentage of women that met the physical activity guidelines were classified according to three categories: (1) sufficient activity to meet the guideline, (2) insufficient activity to meet the guideline, and (3) no activity. Sufficient, insufficient, and no activity are defined for each of the six guidelines in Table 1. Because it is not physically possible to accumulate zero MET minutes of activity throughout the entire week, the “no activity” category was not used for the MET minute definition (guideline 6). 

### 2.6. Statistical Analyses

Descriptive characteristics of the participants are summarized using mean ± standard deviation (SD). Physical activity data were tested for normality prior to any analysis using the D'Agostino-Pearson test and visually analyzed with the use of normality plots and histograms to evaluate the distribution of the data. Total minutes of weekly physical activity according to the parameters of each physical activity guideline are described as medians and interquartile ranges (IQR). The number of women meeting each physical activity guideline is represented graphically and as percentages. All statistical analyses were performed in Med Calc version 12.3 (MedCalc Software, Mariakerke, Belgium). 

## 3. Results and Discussion

### 3.1. Results: Participant Characteristics

On average, the women were 29 ± 4.2 years old, primarily Caucasian (94%, *n* = 66), had 1 ± 1.4 previous pregnancy (not including the current pregnancy), had 0.8 ± 1.3 live births, and had a pre-pregnancy BMI of 24.9 ± 4.7 kg *·* m^2^. 

### 3.2. Results: Minutes of Moderate-Vigorous Physical Activity

Results from the D'Agostino-Pearson test and normality plots revealed a nonnormal distribution (week 18: *P* < 0.05 and week 35: *P* < 0.0001) when all guidelines were applied to the data except weekly accumulated MVPA at week 35 (guideline 1) (*P* = 0.07) and MET minutes at both week 18 and 35 (guideline 6) (*P* = 0.52 and *P* = 0.44, resp.). Minutes of MVPA as assessed by each guideline are reported in Table 2. The very small differences in minutes of activity between MVPA bouts (wk 18 : 141 min; wk 35 : 118 min) and M2VPA bouts (wk 18 : 145 min; wk 35 : 125 min) are explained by the very small amount of vigorous activity performed by this population. 

### 3.3. Results: Adherence to Physical Activity Guidelines

**Figure 1:**
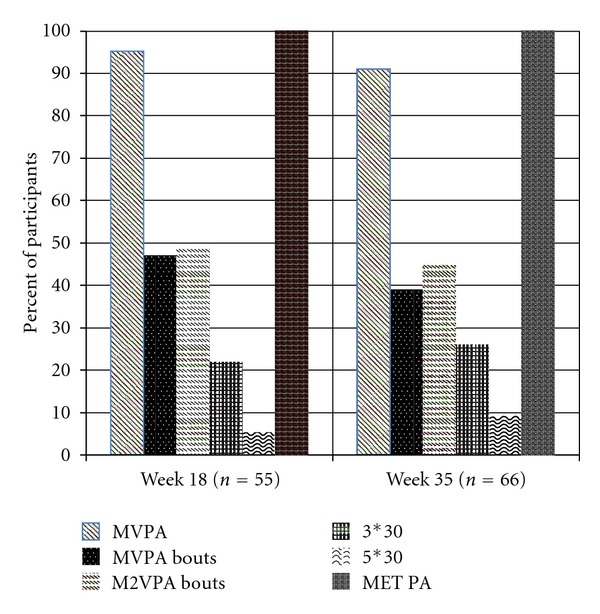
Percentage of pregnant women meeting physical activity guidelines. MVPA: moderate-vigorous physical activity accumulated throughout the week; MVPA bouts: moderate-vigorous physical activity performed in bouts of at least 10 minutes; M2VPA bouts: moderate-vigorous physical activity performed in bouts of at least 10 minutes with 1 minute of vigorous physical activity equivalent to 2 minutes of moderate activity; 3∗30: at least 3 sessions of moderate-vigorous physical activity sustained for at least 30 minutes; 5∗30: at least 5 sessions of moderate-vigorous physical activity sustained for at least 30 minutes; MET PA: total accumulation of weekly MET minutes.

The percentage of women meeting physical activity guidelines ranged from 5% to 100% at week 18 and 9% to 100% during week 35 (see Figure 1). The percentage of women participating in “no activity” ranged from 0 to 42% at week 18 and 0 to 44% at week 35 (see Table 3). All women met the MET minute guideline of at least 500 MET minutes of accumulated weekly activity at both week 18 and week 35. Guidelines requiring 3 or 5 sustained bouts of 30 minutes (guidelines 4 and 5) were met by the fewest number of women at both week 18 (22% and 5%, resp.) and week 35 (26% and 9%, resp.). 

### 3.4. Discussion

The current study revealed wide ranges in both amount of physical activity performed during pregnancy and percentage of women meeting physical activity guidelines depending upon which guideline is used and how guidelines are interpreted. The evaluation of multiple guidelines, the use of an objective monitor that has been evaluated for use in pregnancy, and the longitudinal design contribute novel research findings regarding the assessment of prenatal physical activity. The terms “exercise” and “physical activity” are commonly used interchangeably despite having distinct definitions [27]. Guidelines set forth by ACOG encourage exercise [2] and the Department of Health and Human Services recommends physical activity [8]. The considerable variability in the literature as to how to define recommended activity during pregnancy has contributed, among other factors, to multiple inconsistent conclusions as to whether or not physical activity and/or exercise is an effective way to reduce adverse pregnancy outcomes such as excess gestational weight gain, gestational diabetes, and preeclampsia. 

One potential reason for the inconsistencies in defining physical activity during pregnancy and the prevalence of women meeting physical activity guidelines in the literature is the subtle differences in the wording of the ACOG exercise guideline [2]. The abstract of the guideline recommends that pregnant women engage in 30 minutes or more of moderate exercise a day on most, if not all, days of the week. The opening paragraph of the ACOG guideline [2] then states that pregnant women can adopt the 1995 Centers for Disease Control and Prevention (CDC) and American College of Sports Medicine's (ACSM) recommendation for exercise for nonpregnant adults [27]. This recommendation is to accumulate 30 minutes or more of moderate exercise on most, if not all, days of the week. In contrast to the ACOG guidelines, the 1995 CDC/ACSM recommendation did not explicitly state a minimum length of time that activity should be sustained to count towards meeting the suggested 30 minutes a day. In the 2007 ACSM/American Heart Association's updated recommendation on physical activity and health, bouts of at least 10 minutes were recommended [9]. However, pregnancy guidelines for Americans have not yet adopted this part of the recommendation. 

It is possible that a lack of understanding regarding the maternal and fetal benefits of accumulated total activity versus the benefits of activity sustained for a minimum period of time (e.g., 10, 20, or 30 minutes) has contributed to the discrepancies. It has yet to be determined if pregnancy outcomes differ when activity is evaluated as the total accumulated the activity above a specific threshold compared to activity above a specific threshold sustained for a certain period of time (e.g., 10, 20, or 30 minutes) [12]. Furthermore, it is possible that the appropriate amount of physical activity during pregnancy is outcome specific, similar to physical activity recommendations for nonpregnant adults for reduced risk of premature death, cardiovascular disease, and type 2 diabetes mellitus (150 minutes weekly), mental health benefits (3–5 times/wk for 30–60 minutes), and weight loss or weight maintenance (at least 300 minutes) [8]. 

Recommendations to perform 150 minutes of moderately intense activity, such as the DHHS guidelines, have been adopted for use in pregnancy from nonpregnant physical activity guidelines. This amount of activity was not specifically selected as the recommended amount of weekly activity during pregnancy because of its association with particular health outcomes in pregnant women. Rather, it is recommended as a safe amount of activity assuming a healthy pregnancy with no contraindications. Despite this fact, this amount of activity has been applied as the basis of several prenatal physical activity interventions and may or may not be the appropriate volume to reduce the incidence of adverse pregnancy outcomes. These outcomes include, but are not limited to, excessive gestational weight gain, gestational diabetes, and preeclampsia. As a result, the current literature reports considerable discrepancies as to whether or not the maternal physical activity is an effective approach to prevent such complications [28–34]. 

**Figure 2:**
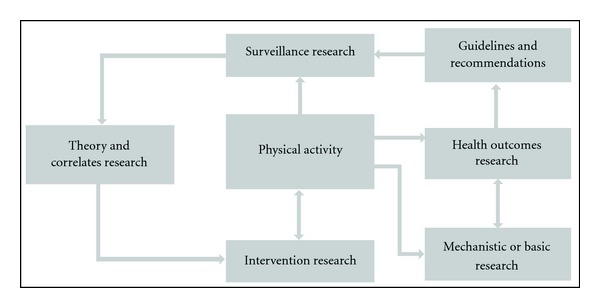
A model of physical activity epidemiology reproduced from [35].

To advance research on physical activity in pregnancy, a more systematic epidemiological approach is needed. Welk outlined a physical activity epidemiology model that shows how different types of physical activity research interact to collectively advance the science [35]. In this model (see Figure 2), *health outcomes research* defines the appropriate volume (duration, frequency, intensity) and type of activity related to specific health benefits for multiple populations all across the life span. This type of research is pertinent to the development of *physical activity guidelines and recommendations* because it focuses on relating the effects of a specific volume of physical activity to multiple indicators of health. The identified volumes of activity associated with particular health outcomes can then be incorporated into public guidelines and recommendations for improved health, or in this case, improved pregnancy outcomes such as reduced excess gestational weight gain and prevalence of gestational diabetes. 

The previously described studies used a variety of methods (both subjective and objective) to assess physical activity data and a variety of definitions to determine the adherence to physical activity guidelines. Additional research is needed to determine the volume and type of physical activity necessary to promote optimal health outcomes for both the mother and the baby. Thus, it is important to note that while these studies may not all be directly comparable to one another, the current study provides evidence to explain some of the inconsistencies in the literature and demonstrates the need for *health outcomes research* to provide pregnancy-specific guidelines for particular maternal and fetal outcomes. Furthermore, it is imperative to use an assessment tool, whether it be subjective or objective, which has been validated for use in pregnant women. The activity monitor used in the current study is a sensitive tool that is effective at capturing physical activity [14] and additionally has been previously evaluated in pregnant women [21]. The monitor was worn 24 hours a day (with the exception of water activities) for a 7-day period. Previous studies have also used accelerometry to assess physical activity during pregnancy, but data were commonly processed for partial days (e.g., 8 or 10 hours of wear time over a 24-hour period) [11, 36]. Thus, while amounts of accumulated physical activity by the participants in this study may be higher than some previous reports [11, 13, 18, 36], the accumulated moderate activity data shown here represent common activity for pregnant women accrued in the course of daily living because a tool previously evaluated in pregnant women was used, data were appropriately adjusted for previously reported overestimation of the assessment tool when used in this population, and participants wore the monitor nearly for 97% of the 7-day monitoring period. 

A limitation of the current study is the differing number of women analyzed at week 18 and week 35 of pregnancy. SWA data was not available for 11 women at week 18 and some of these women may have exercised during their pregnancy. Additionally, participation in a prenatal aqua aerobics class is common in the local community. Due to the large popularity of this class, there is a waiting list to participate and women are typically able to begin participating in this class near weeks 20–22 of pregnancy. Since our observation of activity occurred near week 18 of pregnancy, it is likely that some women were not participating in this class at week 18 but were by the time physical activity was assessed at week 35. Therefore, the higher percentage of women meeting guidelines 4 and 5 (3 or 5 sessions of 30 minutes) at week 35 than week 18 is likely due to the combination of these two circumstances. Lastly, physical activity was defined in the current study by any activity of at least moderate intensity. It was not categorized by leisure time activity versus volitional exercise. This allowed all physical activity of at least moderate intensity to be detected by an objective monitor and eliminated the potential for the recall bias or the incomplete reporting of the physical activity in the physical activity record. It also provided the advantage to capture all accumulated, shorter bouts of at least moderate intensity rather than just longer sustained bouts of activity. 

## 4. Conclusions

The amount of time pregnant women spend in moderate-vigorous physical activity or volitional exercise varies drastically depending upon what guideline is used. Previous reports regarding the prevalence of physical activity during pregnancy have ranged from as low as 3% to as high as 78%. The large range is due in part to the multiple different guidelines that have been used in these studies and the interpretation of these guidelines (e.g., accumulated activity and activity in bouts). Furthermore, interventions have used these guidelines as a target level of physical activity for women to engage in during pregnancy in order to promote specific pregnancy outcomes (e.g., healthy gestational weight gain and improved glucose tolerance) and have provided inconsistent results. Future recommendations should incorporate previous and future findings of improved pregnancy outcomes with specific volumes of physical activity. Additionally, further research is warranted to identify positive pregnancy outcomes associated with clearly identified definitions of physical activity and/or exercise.

## Figures and Tables

**Table 1:** Definitions of physical activity guidelines and activity categorizations.

PA guideline	MVPA	MVPA bouts	M2VPA bouts	3 ∗ 30	5 ∗ 30	MET PA
Sufficient	≥150 min accumulated MVPA	≥150 min MVPA performed in ≥10 min bouts	≥150 min MVPA performed in ≥10 min bouts with 1 min VPA = 2 min MPA	≥3 sessions MVPA sustained for ≥30 mins	≥5 sessions MVPA sustained for ≥30 mins	≥500 MET min

Insufficient	1–149 min accumulated MVPA	1–149 min MVPA performed in ≥10 min bouts	1–149 min MVPA performed in ≥10 min bouts with 1 min VPA = 2 min MPA	1-2 sessions MVPA sustained for ≥30 mins	1–4 sessions MVPA sustained for ≥30 mins	1–499 MET minutes

None	0 min accumulated MVPA	0 min MVPA performed in ≥10 min bouts	0 min MVPA performed in ≥10 min bouts with 1 min VPA = 2 min MPA	0 sessions MVPA sustained for ≥30 mins	0 sessions MVPA sustained for ≥30 mins	N/A

Sufficient activity was defined as enough activity to meet the guideline. MVPA: moderate-vigorous physical activity; M2VPA: 1 minute of vigorous physical activity is equivalent to 2 minutes of moderate activity. N/A: The “none” category is not applicable to this guideline.

**Table 2:** Weekly minutes spent in moderate-vigorous physical activity according to multiple guidelines.

	Week 18	Week 35
	Median min · wk^−1^ (IQR) *n* = 55	Median min · wk^−1^ (IQR) *n* = 66
MVPA	455 (351–585)	468 (240–644)
MVPA bouts	141 (79–199)	118 (31–257)
M2VPA bouts	145 (86–221)	125 (32–268)
3 ∗ 30	1 bout (0–2)	1 bout (0–3)
5 ∗ 30	1 bout (0–2)	1 bout (0–3)
MET PA	10664 (10052–11228)	10433 (9587–11288)

MVPA: moderate-vigorous physical activity accumulated throughout the week; MVPA bouts: moderate-vigorous physical activity performed in bouts of at least 10 minutes; M2VPA bouts: moderate-vigorous physical activity performed in bouts of at least 10 minutes with 1 minute of vigorous physical activity equivalent to 2 minutes of moderate activity; 3 ∗ 30: At least 3 sessions of moderate-vigorous physical activity sustained for at least 30 minutes; 5 ∗ 30: At least 5 sessions of moderate-vigorous physical activity sustained for at least 30 minutes; MET PA: total accumulation of weekly MET minutes.

**Table 3:** Distribution of pregnant women meeting physical activity guidelines.

	Week 18 of pregnancy, *n* = 55 (% (*n*))						Week 35 of pregnancy, *n* = 66 (% (*n*))					
	MVPA	MVPA bouts	M2VPA bouts	3 ∗ 30	5 ∗ 30	MET PA	MVPA	MVPA bouts	M2VPA bouts	3 ∗ 30	5 ∗ 30	MET PA
Sufficient	95 (52)	47 (26)	49 (27)	22 (12)	5 (3)	100 (55)	91 (60)	39 (26)	44 (29)	26 (17)	9 (6)	100 (66)
Insufficient	5 (3)	49 (27)	47 (26)	36 (20)	53 (29)	0	9 (6)	56 (37)	52 (34)	30 (20)	47 (31)	0
None	0	4 (2)	4 (2)	42 (23)	42 (23)	N/A	0	5 (3)	5 (3)	44 (29)	44 (29)	N/A

MVPA: moderate-vigorous physical activity accumulated throughout the week; MVPA bouts: moderate-vigorous physical activity performed in bouts of at least 10 minutes; M2VPA bouts: moderate-vigorous physical activity performed in bouts of at least 10 minutes with 1 minute of vigorous physical activity equivalent to 2 minutes of moderate activity; 3 ∗ 30: at least 3 sessions of moderate-vigorous physical activity sustained for at least 30 minutes; 5 ∗ 30: At least 5 sessions of moderate-vigorous physical activity sustained for at least 30 minutes; MET PA: total accumulation of weekly MET minutes.
